# Live cell imaging reveals extensive intracellular cytoplasmic colonization of banana by normally non-cultivable endophytic bacteria

**DOI:** 10.1093/aobpla/plu002

**Published:** 2014-01-16

**Authors:** Pious Thomas, Aparna Chandra Sekhar

**Affiliations:** Division of Biotechnology, Indian Institute of Horticultural Research, Hessarghatta Lake, Bangalore 560 089, India

**Keywords:** Bacterial endophytes, Brownian motion, confocal laser scanning microscopy, epi-fluorescence microscopy, micropropagation, *Musa* sp., non-cultivable bacteria, plant tissue culture, triphenyl tetrazolium chloride.

## Abstract

It is generally believed that endophytic microorganisms are intercellular inhabitants present in either cultivable or non-cultivable form primarily as root colonizers. The objective of this study was to determine whether the actively mobile micro-particles observed in the intracellular matrix of fresh tissue sections of banana included endophytic bacteria. Tissue sections (50–100 µm) from apical leaf sheaths of surface-disinfected suckers (cv. Grand Naine) displayed ‘Brownian motion’-reminiscent abundant motile micro-particles under bright-field and phase-contrast (×1000), which appeared similar in size and motility to the pure cultures of endophytes previously isolated from banana. Observations on callus, embryonic cells and protoplasts with intact cell wall/plasma membrane confirmed their cytoplasmic nature. The motility of these entities reduced or ceased upon tissue fixation or staining with safranin/crystal violet (0.5 % w/v), but continued uninterrupted following treatment with actin-disrupting drugs, ruling out the possibility of micro-organelles like peroxisomes. Staining with 2,3,5-triphenyl tetrazolium chloride (TTC) confirmed them to be live bacteria with similar observations after dilute safranin (0.005 %) treatment. Tissue staining with SYTO-9 coupled with epi-fluorescence or confocal laser scanning microscopy showed bacterial colonization along the peri-space between cell wall and plasma membrane initially. SYTO-9 counterstaining on TTC- or safranin-treated tissue and those subjected to enzymatic permeabilization revealed the cytoplasmic bacteria. These included organisms moving freely in the cytoplasm and those adhering to the nuclear envelope or vacuoles and the intravacuolar colonizers. The observations appeared ubiquitous to different genomes and genotypes of banana. Plating the tissue homogenate on nutrient media seldom yielded colony growth. This study, supported largely by live cell video-imaging, demonstrates enormous intracellular colonization in bananas by normally non-cultivable endophytic bacteria in two niches, namely cytoplasmic and periplasmic, designated as ‘Cytobacts’ and ‘Peribacts’, respectively. The integral intracellular association with their clonal perpetuation suggests a mutualistic relationship between endophytes and the host.

## Abstract (precis)

The general understanding about endophytic microorganisms is that they are inhabitants in the free intercellular spaces primarily in roots. This study uncovers extensive cytoplasmic colonization by endophytic bacteria in banana shoot-tissue which *prima-facie* appeared like ‘Brownian movement’. Live cell imaging on tissue sections, callus, cell suspensions and protoplasts directly and after vital / SYTO-9 staining has brought out two intracellular niches, namely cytoplasmic and periplasmic. Designated as ‘Cytobacts’ and ‘Peribacts’, these organisms appeared to be normally not amenable for cultivation and thus possibly escaped the attention of biologists. The observations here open the way to study these intracellular entities distinct from micro-organelles.

## Introduction

The term endophyte denotes microorganisms including bacteria and fungi that colonize plants internally without any apparent adverse effects on the host (Hallmann 2001; Bacon *et al.* 2002; Reinhold-Hurek and Hurek 2011). Endophytic bacteria are known to be associated with diverse plant species and organs (Hallmann *et al.* 1997; Rosenblueth and Martínez-Romero 2006; Thomas *et al.* 2007; Sun *et al.* 2008). As per the earlier understanding, endophytes were considered to colonize the host predominantly in roots (Hallmann 2001; Bacon *et al.* 2002; Bulgarelli *et al.* 2012), and be present in low abundance compared with the general rhizoplane colonizers (Hallmann 2001; Compant *et al.* 2010). Studies adopting microscopic examination of tissue homogenate, on the other hand, have indicated that the organisms are present in substantial numbers; their general non-cultivability being the prime reason for them escaping the attention of biologists (Thomas and Soly 2009; Thomas 2011; Thomas and Reddy 2013).

The intra-tissue inhabitation in close association with the host with possible mutualistic effects makes the endophytes important in plant biology (Rosenblueth and Martínez-Romero 2006; Compant *et al.* 2010; Reinhold-Hurek and Hurek 2011; Thomas 2011). Pyrosequencing and metagenome-based studies have shown a considerable diversity of root endophytes with substantial insights into their functions (Wang *et al.* 2008; Bulgarelli *et al.* 2012; Lundberg *et al.* 2012; Sessitsch *et al.* 2012). The information on the extent and regions of tissue colonization assumes importance in relation to functional genomics and while deciphering the roles of endophytes.

Conventional approaches for tissue localization of endophytes include direct tissue fixation and staining (Hallmann *et al.* 1997), transmission electron microscopy (TEM) (Gyaneshwar *et al.* 2001; Sattelmacher 2001) or fluorescent *in situ* hybridization (Pirttilä *et al.* 2000; Compant *et al.* 2011). Such studies have indicated tissue colonization predominantly in the intercellular spaces in roots and xylem tissue. Confocal imaging with the use of fluorescently labelled endophytes facilitates studying the colonization pattern of selected organisms and their possible functions. Such studies have reinforced the understanding about the entry of endophytes through root hairs or root epidermis, traversal through cortical parenchyma and subsequent upward movement through xylem (Compant *et al.* 2005, 2008; Prieto *et al.* 2011). Some reports have suggested intracellular colonization as in the case of shoot meristem in Scotch pine (Pirttilä *et al.* 2000) or *in vitro* cultures of peach palm (de Almeida *et al.* 2009). Any colonization in the cytoplasmic matrix has considerable implications since the organisms would be surviving similar to cell organelles with an interactive effect on cell machinery and function.

This study was undertaken as a follow-up to earlier observations with banana indicating the ubiquitous association of endophytic bacteria in field-derived shoot-tip explants and *in vitro* cultures in substantial numbers predominantly in non-cultivable form as observed with the tissue homogenate (Thomas *et al.* 2008*a*, *b*; Thomas and Soly 2009). Subsequent studies employing Live-Dead bacterial staining kit components SYTO-9 and propidium iodide on fresh shoot tissue of banana genotypes supported by TEM have demonstrated substantial bacterial colonization in the confined peri-space between plasma membrane and cell wall (Thomas and Reddy 2013). Conversely, simple microscopic examination of fresh leaf-sheath sections under bright-field showed actively mobile particles in the cell lumen, their motility *prima facie* suggested ‘Brownian motion’. However, they appeared similar in size and motility to the cells of some of the endophytes isolated from banana, in particular the coccus-shaped non-filamentous Actinobacteria (Thomas *et al.* 2008*b*; Thomas and Soly 2009). These intracellular motile units, however, were not detected with SYTO-9, which otherwise had stained the bacterial cells in the peri-space (Thomas and Reddy 2013). Vital staining using 2,3,5-triphenyl tetrazolium chloride (TTC), which helps in the detection of live endophytic bacteria (Bacon *et al.* 2002; Thomas 2011), have indicated the staining of some of the motile and non-motile units of intra-tissue, suggesting that they are live bacteria. With vital and fluorescent bacterial staining on tissue sections as well as callus, cell suspension and protoplast cultures, the present study, primarily through live cell video-imaging, demonstrates the extensive intracellular colonization in the cytoplasmic niche by bacterial endophytes in native banana plants in addition to the recently documented periplasmic colonization.

## Methods

### Plant material

Field-grown suckers of banana cultivars Grand Naine, Robusta and Dwarf Cavendish (AAA genome), Rasthali, Mysore, Ladies Finger (AAB), Udhayam, Monthan (ABB) and Ney Poovan (AB) were used in the investigations. Shoot tips (12–15 mm) gathered from 3- to 4-month-old field suckers after removing the external leaf sheaths were used in microscopic studies either directly or after surface disinfection treatments as per Thomas *et al.* (2008*a*). In addition, micropropagated cultures of Grand Naine, Robusta and Dwarf Cavendish in different subculture passages, grown on MS-based tissue culture medium as described earlier (Thomas *et al.* 2008*a*), and the acclimatized plants *in vivo* were also employed. Cultivar Grand Naine was used in different studies with or without tissue fixation in formalin–phosphate-buffered saline (Thomas and Reddy 2013). Callus cultures were initiated from floral apices of Grand Naine and Robusta and were maintained as per Strosse *et al.* (2003), or were obtained from the National Research Centre for Banana (NRCB; Tiruchirapally, Tamil Nadu, India) as embryonic cell suspension (ECS) cultures. Reference to a culture or cell in this report pertains to banana unless specified as bacterial culture or cell; the latter are also referred to as motile particles. All the tissue culturing and sample preparation work was carried out under stringent sterility checks as described elsewhere (Thomas and Reddy 2013).

### Bright-field microscopy on tissue sections from field shoot tips and *in vitro* cultures

Thin tissue sections (∼50–100 µm) were prepared from the inner leaf sheaths of suckers (Grand Naine) using a fine razor blade over a sterilized glass slide (Thomas and Reddy 2013) and were examined under ×1000 bright-field or phase-contrast after mounting in sterile distilled water. Live images and videos on tissue sections in the horizontal plane (*x*–*y*) or with vertical fine focusing (*x*–*y*–*z*) were captured over 20–30 s time (*t*) using a Leica DM2000 optical microscope with a DFC-295 camera and Leica Application Suite (LAS) software (Leica Microsystems CMS GmbH, Wetzlar, Germany). The observations were extended to Grand Naine suckers gathered from different garden sources (20 suckers) employing shoot tips with or without surface sterilization. Micropropagated cultures as well as acclimatized plants of Grand Naine established in autoclaved planting medium were also taken up. The observations were validated with eight other genotypes mentioned above.

### TTC staining and bright-field microscopy on tissue sections

Leaf sheaths from surface-sterilized shoot-tip explants were separated and soaked in TTC (1.5 g L^−1^)–malic acid (625 mg L^−1^) solution in 0.05 M potassium phosphate buffer (pH 7.0) as per Bacon *et al.* (2002) and Thomas (2011) as a vital bacterial staining technique where the bacterial cells are expected to stain pink or red. After 1–5 days, thin (∼50–100 µm) tissue sections were prepared from (i) the inner leaf sheaths, (ii) the sheath base and (iii) the basal corm tissue using a fine razor blade under aseptic conditions. Prior to tissue sampling, leaf sheaths were rinsed repeatedly to remove any external organisms. This was repeated post-sectioning. Tissue sections were mounted in sterile water and still images and live videos were captured as above.

### Bright-field microscopy after crystal violet, safranin or methylene blue staining of tissue sections

Bacterial staining employing TTC required a minimum of overnight incubation in TTC–malic acid buffer. To avoid the chance of any bacterial multiplication occurring during this period, staining of freshly prepared leaf-sheath sections with filter-sterilized bacterial dyes crystal violet and safranin (0.005–0.1 % w/v), which stain pure bacterial cultures violet and red, respectively, was tried in addition to the nuclear stain methylene blue (0.05–0.5 %).

### Bright-field microscopy on callus and cell suspension cultures

The callus stocks bear easily dissociable loose cells with the chance of observing isolated cells with intact cell wall and ascertaining the intracellular attributes. The cells from the callus stocks of Grand Naine and Robusta were dispersed on a clean microscopic slide and were rinsed repeatedly to remove any external bacteria. After mounting in sterile water, they were examined under bright-field and phase-contrast directly as well as after a brief (2–3 min) staining with diluted (0.005 %) safranin. Similarly ECS cultures of Grand Naine, Robusta and Rasthali obtained from the NRCB were examined under bright-field with or without safranin staining.

### Bright-field microscopy on protoplasts

To facilitate the viewing of cells in isolation, protoplasts were prepared from callus and ECS cultures and from dark-incubated leaf tissue adopting the protoplast isolation procedure of Wu *et al.* (2009) with some modifications. Tissues were incubated in filter-sterilized protoplast isolation buffer containing 1.0 % (w/v) cellulase (Sigma, St Louis, USA) and 0.25 % pectinase (HiMedia Life Sciences, Mumbai, India) at 26–28 °C for 4 h at 60 rpm followed by six rinses to remove the debris and extraneous bacterial cells. Host cells were separated by gently pressing with a cover glass over the microscope slide and then rinsed repeatedly to remove any external bacteria. Protoplasts were examined directly or after mounting in 0.005 % safranin under bright-field microscopy (×1000 magnification).

### Epi-fluorescence microscopy on tissue sections, callus and protoplasts with SYTO-9

Fresh tissue sections gathered from shoot-tip leaf sheaths were covered with SYTO-9 (6 μM in water) from the Live–Dead bacterial staining kit L13152 (Molecular Probes^®^, Life Technologies, New York, USA) which stains the live bacterial cells green (Thomas and Reddy 2013). The sections were examined after 10–20 min using a Leica DM-LB2 epi-fluorescence microscope with a DFC320 digital live camera (Leica Microsystems CMS GmbH, Wetzlar, Germany). The images were captured under a ×100 water-immersion objective with GFP filter in blue excitation and processed as described elsewhere (Thomas and Reddy 2013). Additionally, SYTO-9 staining of leaf and corm tissue sections after a brief exposure to safranin and of the sections prepared from TTC-soaked tissue was undertaken. In addition, callus and ECS cultures and protoplasts prepared from them as described above were also examined after SYTO-9 staining.

### Confocal laser scanning microscopy on tissue sections, callus and protoplasts with SYTO-9

Confocal laser scanning microscopy (CLSM) on freshly prepared tissue sections after staining with SYTO-9 was undertaken using a LSM 5 LIVE confocal laser scanning microscope equipped with a 488-nm laser and supported by the LSM software (Carl Zeiss Inc., Jena GmbH, Germany). The images were captured in *x*–*y*–*z* and time series for 25–30 s and processed as described elsewhere (Thomas and Reddy 2013). Additionally, callus, embryonic cultures and protoplasts were also examined after SYTO-9 staining under CLSM. The freely available software Image J was used to generate avi files from time-lapse and *z*-stacks as discussed elsewhere (Thomas and Reddy 2013).

### Distinguishing intracellular bacteria from plant microbodies

Plant microbodies including peroxisomes, glyoxisomes, lysosomes and Golgi vesicles are known to show motility in the intracellular matrix and have a similar size and shape to coccus-shaped bacteria but they do not bear DNA (Mathur *et al.* 2002; Akkerman *et al.* 2011; Hu *et al.* 2012). To discriminate the intracellular organisms from plant microbodies, which move in the cellular matrix along the actin filaments, callus stocks, ECS and protoplasts were treated with the actin-depolymerizing drug 2,3-butanedione monoxime (15, 30 or 60 mM for up to 2 h) or latrunculin B (1 µM) to see whether the intracellular bacterial motility was disrupted as for plant peroxisomes (Jedd and Chua 2002; Mathur *et al.* 2002). Use of the microtubule-disrupting chemical nocodozole (3.3 µM) was considered as peroxisomes move on microtubules in animal cells (Jedd and Chua 2002).

### Plating the tissue homogenate from shoot-tip explants

Shoot-tip explants derived from field suckers of Grand Naine (20 suckers) after surface sterilization were used for assessing bacterial colonization. Tissue homogenate (100 mg mL^−1^) was prepared and plated on nutrient media and monitored for colony growth (Thomas and Soly 2009).

## Results

### Bright-field microscopy on tissue sections

The results presented here pertain to cv. Grand Naine unless specified otherwise. Tissue sections from field-derived shoot tips and micropropagated stocks showed plastids and mitochondria under phase-contrast and bright-field as stationary objects of roughly 2–5 µm, as documented elsewhere (Thomas and Reddy 2013). An *x*–*y*–*z* bright-field scan showed actively motile micro-particles (≤1 µm) in the cellular matrix in both leaf sheath and corm portions **[see Supporting Information—Movie 01]**. These included small rods and cocci. *Prima facie,* their motility appeared like ‘Brownian motion’. A comparison with the pure cultures of endophytic bacteria previously isolated from banana indicated similarity in size, shape and motility to these intracellular particles **[see Supporting Information—Movie 02]**. The motile units in fresh tissues sections varied in their abundance from cell to cell or part of the tissue segment, ranging from high numbers towards the base of the leaf sheath to low abundance in the upper region. Variation in distribution was also observed from the exterior to the interior of the leaf sheaths with no obvious ones in some cells or tissue parts. The motility of these intracellular particles, seen in different cell layers during fine focusing, became more obvious a short while after the photo-illumination.

The above observations held good with a series of Grand Naine suckers gathered from different gardens and with the other genotypes, namely, Mysore **[see Supporting Information—Movie 03]**, Udhayam, Ladies Finger, Ney Poovan, Monthan, Robusta, Dwarf Cavendish and Rasthali (video not presented). Variation from genotype to genotype and from cell to cell in a genotype in the type or the extent of mobile units was observed. It appeared that the cells that were cut through or disturbed displayed them in abundance, while in some fields they were not obvious or remained static. Application of gentle pressure over the cover-glass rendered such cellular granules motile.

### TTC staining of tissue and bright-field microscopy

**Figure 1.**
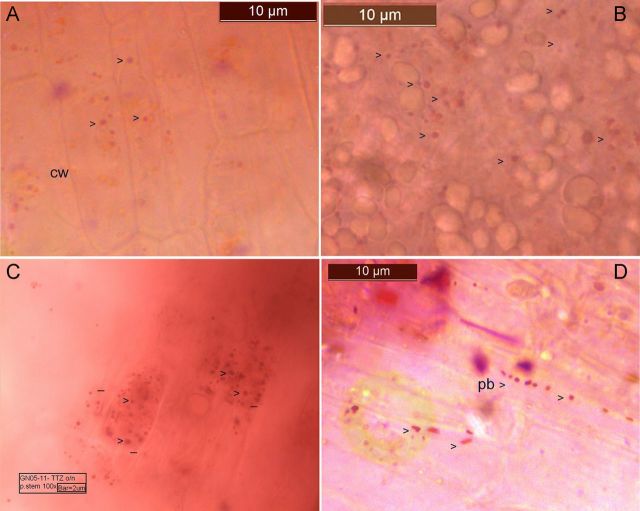
Bacterial colonization in banana ‘Grand Naine’ *in vitro* cultures demonstrated through TTC staining under bright-field. Tissue from surface-sterilized *in vitro* plants displaying pink- or red-stained bacteria (arrowheads) along with other cell organelles in the cell matrix of leaf sheaths or corm after incubation in TTC for 1–2 days (A, B) or intense staining (C), and along the peri-space (D) with 4–5 days treatment (cw = cell wall; pb = peri-space bacteria). Bar length is as marked in the figure.

Tissue sections from TTC-treated shoots showed light tissue pigmentation along with pink-stained or unstained micro-particles obviously in the intracellular matrix either as non-motile units or as actively mobile ones in both leaf sheath and corm (Fig. 1A and B) **[see Supporting Information—Movie 04]**. It was also possible to observe non-stained cell organelles like plastids and mitochondria and unstained micro-particles under bright-field and phase-contrast. Some irregular-shaped larger bodies that were stained with TTC were also seen (image not shown). Extended incubation in TTC imparted a deeper staining of motile and smaller particles (Fig. 1C) confirming them to be live bacteria and less likely to be micro-organelles or other cellular inclusions. It was also possible to see the TTC-stained bacteria in the peri-space along the boundary in some cells (Fig. 1D). Not all the motile particles were stained by TTC, and this was in line with the observations on pure cultures of endophytes from banana and as documented with watermelon endophytes (data not presented). Tissue-incubated TTC solution displayed very few bacterial cells compared with the intra-tissue region. 2,3,5-Triphenyl tetrazolium chloride staining of intracellular bacteria held good with shoot tissue from field-grown suckers of other genotypes as well as the micropropagated cultures of ‘Robusta’ and ‘Dwarf Cavendish’ through 5–6 *in vitro* passages. The same was true with the acclimatized ‘Grand Naine’ plants (data not shown).

### Bright-field microscopy after crystal violet, safranin or methylene blue tissue staining

Staining of tissue sections with crystal violet, safranin or methylene blue (0.1–0.5 % w/v) resulted in high tissue pigmentation and an instant loss or a drastic reduction in bacterial motility depending on the stain, the concentration or the duration of treatment. Thereafter it became impossible to identify bacterial cells inside the tissue matrix based on their motility. The same was observed with pure cultures of different endophytes from banana, particularly the Gram-positive organisms. However, the cell nucleus, chloroplast and mitochondria became clearer after a brief staining with the stains at 0.05–0.1 % (data not presented). Use of dilute safranin (0.005 %) retained bacterial motility, allowing their detection in the intracellular matrix, particularly in the peripheral thin region of the sections **[see Supporting Information—Movie 05]**.

### Bright-field microscopy on callus and cell suspension cultures

**Figure 2.**
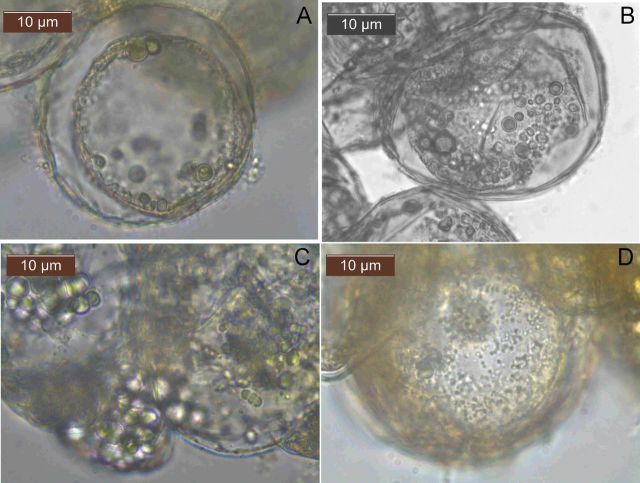
Four different cell types observed with the callus and embryogenic cell cultures of banana cv. Grand Naine under phase-contrast microscopy (×1000) with respect to density of organelles or cellular inclusions: (A) displaying a thick central area of thick inclusions and a peripheral clear area, (B) showing abundant organelles or inclusions distributed in the cell lumen, (C) organelles scattered in the cell matrix with obvious vacuoles or free spaces, and (D) those not showing any obvious large cellular inclusions (scale bar = 10 µm).

Cells from callus and those from ECS with intact cell wall showed four different cell types as per phase-contrast microscopy with respect to the density of organelles or cellular inclusions, namely, (i) those displaying a thick central area of thick inclusions and a peripheral clear area, (ii) those showing abundant organelles or inclusions distributed in the cell lumen, (iii) organelles scattered in the cell matrix with obvious vacuoles or free spaces, and (iv) those not showing any obvious large cellular inclusions (Fig. 2). The above cell types showed the relative abundance of motile bacteria in the cell lumen in the reverse order, i.e. in enormous numbers in the fourth type with a predominance of cocci, and seldom or occasional ones in the first group. Observations with cell cultures, however, confirmed the intracellular nature of bacterial association **[see Supporting Information—Movie 06]**. Microscopy on endophytic bacteria isolated from banana suggested that organisms like *Staphylococcus* sp. and *Micrococcus* sp. possessed cells of similar size, shape and motility as observed with the host cells. The observations on cell cultures were validated with ECS directly and after staining with dilute safranin, which clearly showed their intracellular nature.

### Bright-field microscopy on protoplasts

Clusters of cells from embryonic cultures could be separated following treatment with cellulase and pectinase (2–4 h) yielding single cells or groups of loosened cells with clear cell boundaries confirming the cytoplasmic bacterial inhabitation **[see Supporting Information—Movie 07]**. Similar to callus, four categories of protoplasts were observed with respect to the density of intracellular inclusions and the extent of bacterial colonization as discussed above. Examination of dilute safranin-stained protoplasts showed intravacuolar organisms besides cytoplasmic bacteria **[see Supporting Information— Movie 08]**.

### Distinguishing intracellular bacteria from plant microbodies

Tissue sections treated with the actin-depolymerizing drug 2,3-butanedione monoxime or latrunculin B continued to display intracellular micro-particle motility even after 2–4 h of exposure at the different levels tried. The same appeared to be true for the sections treated with the microtubule-disrupting chemical nocodozole. Embryonic cell cultures as well as protoplast preparations of ‘Grand Naine’ treated with 2,3-butanedione monoxime, latrunculin B or nocodozole also continued to display the intracellular motility even after 2–4 h of continuous exposure. This endorsed the view that the mobile units were unlikely to be peroxisomes or other microbodies. Other major organelles, namely plastids and mitochondria, were much larger in size (2–6 µm) and they could be observed directly under bright-field and phase-contrast.

### Epi-fluorescence microscopy on tissue sections, callus and protoplasts with SYTO-9

**Figure 3.**
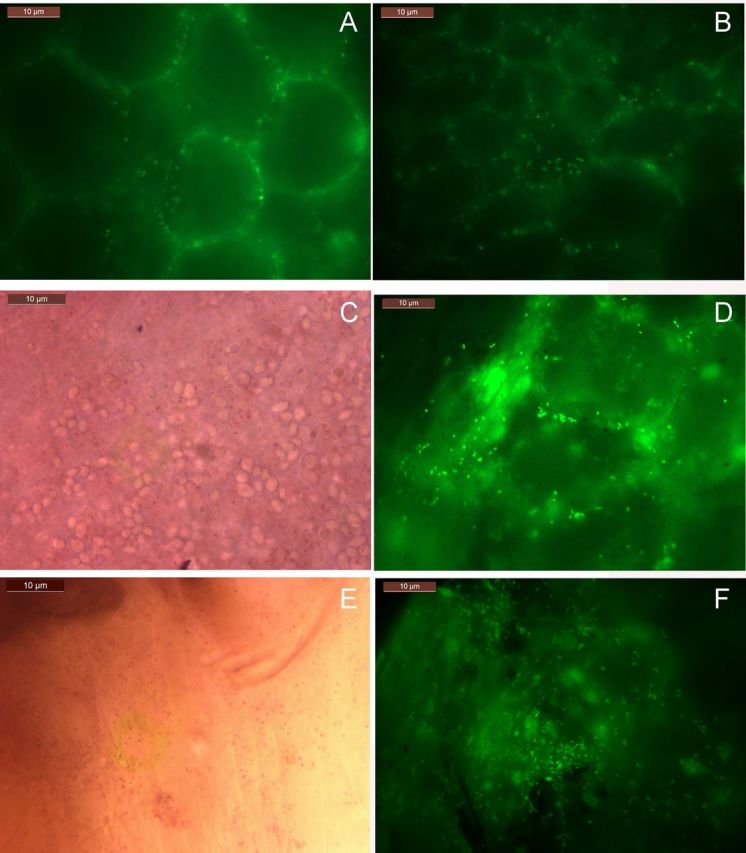
Fresh tissue sections stained with SYTO-9 showing peri-space bacteria along the cell boundary (A) including some in the intracellular space (B), tissue section briefly stained with safranin (0.005 %) showing up pink-stained (C) and green-fluorescing intracellular bacteria upon counterstaining with SYTO-9 (D), and TTC-treated tissue showing red staining of bacteria (E) and their green fluorescence upon counterstaining with SYTO-9 (F).

Fresh tissue sections stained with SYTO-9 showed bacteria along the cell boundary as per the earlier observations on peri-space colonizers (Fig. 3A and B). Tissue sections exposed to brief safranin treatment showed intracellular bacteria upon counterstaining with SYTO-9, which suggested that safranin facilitated the passage of the fluorophore through the plasma membrane (Fig. 3C and D). 2,3,5-Triphenyl tetrazolium chloride-treated tissue also showed fluorescing intracellular bacteria upon staining with SYTO-9 (Fig. 3E and F). SYTO-9 staining of TTC-stained motile particles under bright-field endorsed the view that the TTC-positive motile units did indeed constitute bacteria. Other DNA-containing organelles, namely plastids and mitochondria, were not detected with the direct SYTO-9 staining, but became evident following a permeabilization step with Triton X-100 (0.1 %) for 1 h (data not shown). Tissue sections and cell cultures that were subjected to cellulase + pectinase treatment displayed the staining of intracellular bacteria and the nucleus (data not shown) suggesting that the enzyme treatment facilitated the intracellular entry of the dye and the detection of cytoplasmic bacteria.

### Confocal laser scanning microscopy on tissue sections, callus and protoplasts with SYTO-9

**Figure 4.**
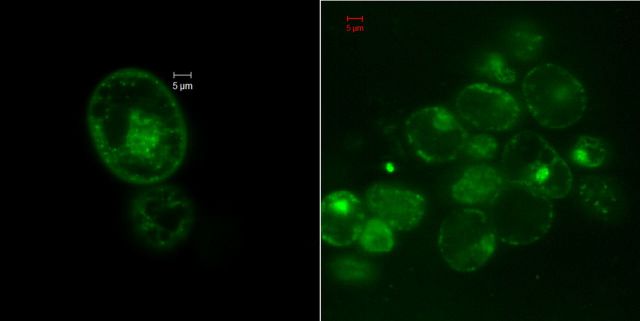
Embryonic cell culture of banana cv. Grand Naine treated with SYTO-9 displaying bacteria as green-fluorescing spots in the cytoplasm and along the peri-space with some bacterial adhesion to the nucleus with confocal laser scanning microscopy (63× objective).

**Figure 5.**
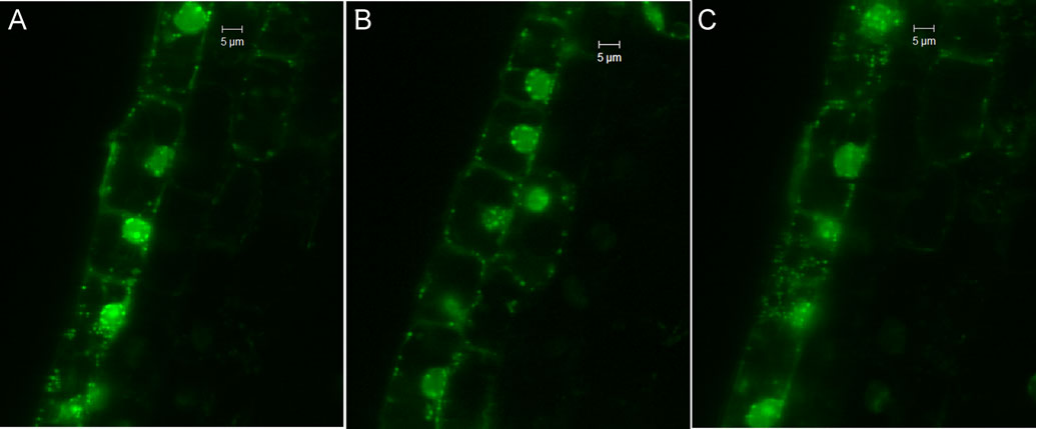
Tissue segment from banana leaf-sheath stained with SYTO-9 after a 1-h permeabilization treatment with cellulase and pectinase displaying abundant bacteria as green-fluorescing spots along the peri-space, inside the cytoplasm and also adhering to the nuclear envelope in different *z*-stacks with confocal laser scanning microscopy (63× objective).

Confocal imaging on fresh tissue sections and callus cells after SYTO-9 treatment displayed the staining of peri-space bacteria as per the earlier observations documented by Thomas and Reddy (2013). The ECS cultures, however, showed staining of both peri-space and cytoplasmic bacteria (Fig. 4) including actively motile ones in different cell layers in the *x*–*y*–*z* scan **[see Supporting Information—Fig. S1 and Movie 09]**. Tissue sections that were subjected to permeabilization treatment with cellulase + pectinase (1 h) showed the staining of abundant cytoplasmic bacteria in the *x*–*y* plane besides the peri-space organisms in different cell layers in the *z-*stack **[**Fig. 5; **see** **Supporting Information—Movie 10****]**. The confocal time-scans did not give a real-time picture upon conversion to video/avi with Image J. The frame speed in the converted files appeared much faster than the real-time situation.

### Plating the tissue homogenate

Tissue homogenate plated on nutrient agar or trypticasein soy agar showed colony growth in 20 % of the surface-sterilized shoot tips and 35 % of samples gathered without surface sterilization with very few colonies (10^2^–10^3^ g^−1^ tissue). However, the tissue homogenate displayed abundant motile bacterial cells under phase-contrast, suggesting that the organisms were largely non-cultivable.

## Discussion

The present study reveals the widespread prevalence of intracellular bacteria in banana predominantly in non-cultivable form, which possibly has evaded the attention of plant biologists. The findings came about as a follow-up of the observations on intracellular motile particles noticed in fresh tissue sections of banana shoot issue. In the recent past, bacterial colonization in the confined peri-space between the cell wall and plasma membrane was documented in different banana genotypes (Thomas and Reddy 2013). Put together, the observations suggest two niches of intracellular bacterial colonization in bananas, namely cytoplasmic inhabitants and periplasmic colonizers with an enormous number of organisms in both niches. Investigations with callus and cell suspension cultures of grape, barley and medicinal species (*Catharanthus roseus, Ajuga reptans, Fallopia sachelensis*, *F. japonica* and *Polygonum cuspidatum*) probing whether the long-term actively maintained ‘supposedly sterile’ cultures harboured any endophytic bacteria have also indicated extensive bacterial colonization in cytoplasmic and periplasmic niches, and the terms ‘Cytobacts’ and ‘Peribacts’ have been proposed to address the organisms that inhabit the cytoplasmic niche and periplasmic niche, respectively (P. Thomas and C. M. Franco, unpubl. res.).

The previous study employing Live-Dead bacterial staining on banana tissue sections showed only the peri-space colonization (Thomas and Reddy 2013). This appeared to be due to the inability of this label to pass through the intact plasma membrane. Enzymatic cell permeabilization or tissue treatment with TTC/safranin facilitated the detection of cytoplasmic bacteria by SYTO-9. The same was observed with grape cell and callus cultures where 0.01 % Triton X-100 for 30 min also facilitated the staining of cytoplasmic bacteria (P. Thomas and C. M. Franco, unpubl. res.). Other DNA-containing organelles, namely mitochondria and plastids, were not stained in this study, which also appeared to be linked to the inability of SYTO-9 to pass through the organelle membranes. With grape cell cultures, a cell permeabilization step (0.01 % Triton X-100 for 30 min) facilitated the staining of cytoplasmic bacteria while an extended treatment (0.1 % Triton X-100 for 1 h) stained the plastids and mitochondria (P. Thomas and C. M. Franco, unpubl. res.).

The non-destructive confocal *z*-scanning on tissue sections across different cell layers and the observations with callus, cell and protoplast cultures with integral cell wall/plasma membrane confirmed the true intracellular nature of bacterial associates. Staining by SYTO-9, which targets DNA, and the sustained intracellular particulate motility in the presence of actin-disrupting drugs indicated that the chances of them being microorganelles were remote as these organelles do not contain DNA and their motility is drastically affected by such drugs (Jedd and Chua 2002; Mathur *et al.* 2002, 2012; Akkerman *et al.* 2011; Hu *et al.* 2012). Variation in the distribution and type of bacteria in different cells, tissues or genotypes was observed in the present study besides their enormous presence in cells where the internal configuration appeared disrupted. The diversity in the type of organisms isolated from banana and the differences in the extent and the kind of organisms associated with different suckers (Thomas *et al.* 2008*b*; Thomas and Soly 2009) may explain such variations. In studies employing *in vitro* cultures of banana and watermelon some of the associated organisms were activated to cultivation from their non-cultivable state with the release of tissue breakdown products in senescing cultures or with host-tissue extract supplementation indicating their reliance on the host metabolites (Thomas and Soly 2009; Thomas 2011).

Altogether the observations suggested that the plant cells harbour an additional entity in the form of live bacteria besides the normal cell organelles. This is endorsed by the findings with the long-term-maintained cell suspension cultures of grapes, barley and medicinal species mentioned above (P. Thomas and C. M. Franco, unpubl. res.) and a similar report on ‘Bacteriosomes’ in peach palm (de Almeida *et al.* 2009). Our preliminary observations with different plant species also suggested that intracellular bacterial association in shoot tissue is possibly a widespread phenomenon. The non-cultivability of the associated organisms, variation in microscopic approaches employed by different research groups, the high magnification (×1000) required to observe the bacterial cells, the general attribution of intracellular entities as micro-organelles and the common tendency to describe the random particle movement as ‘Brownian motion’, possibly explain why this topic was not pursued vigorously by biologists. Our observations with different bacterial species have shown that ‘Brownian motion’ is a common phenomenon associated with most of the organisms and hence such movements also can be taken as an indicator of the bacterial presence. Although Brownian motion was originally described in plant systems (Brown 1828), the concept did not see as much attention or growth in biology as in physics (Astumian and Hänggi 2002; Hänggi and Marchesoni 2005). The present observations call for more investigations on this topic in biological systems.

The findings in this study have been facilitated with the adoption of live-cell imaging in contrast to the general microscopic approaches using fixed tissue where it was difficult to discriminate the bacterial cells from other cellular constituents or inclusions (Thomas *et al.* 2008*a*; Thomas and Soly 2009). Studies adopting epi-fluorescence or confocal microscopy have often employed fixed tissue or microscopy at lower magnifications, at which level the internal details were not resolved sufficiently. Live cell imaging coupled with micro-videography has proved to be a strong tool to study plant–endophyte associations by demonstrating the abundant bacterial colonization in the cytoplasmic and periplasmic niches (Thomas and Reddy 2013; P. Thomas and C. M. Franco, unpubl. res.). Such approaches have proven very valuable in enhancing our current understanding of cellular organization and functions (Stephens and Allan 2003; Shaw 2006; Mathur *et al.* 2012).

Adoption of cultivation-independent molecular approaches to study bacterial endophytes (Reiter *et al.* 2002; Reiter and Sessitsch 2006) including pyrosequencing (Lucero *et al.* 2011; Bulgarelli *et al.* 2012; Lundberg *et al.* 2012) and metagenomics (Sessitsch *et al.* 2012) have shown enormous bacterial diversity in different plant species. Such studies, however, have not placed much emphasis on providing microscopic evidence on tissue colonization. The present report on intracellular shoot colonizers is to be viewed differently from such colonization commonly documented in the root cortex for rhizobacteria or in legume nodules (Parniske 2000; Cocking *et al.* 2006). The observations here deviate from the general understanding on the extent and regions of tissue colonization by bacterial endophytes, which were considered to be present in fewer numbers, primarily as the inhabitants of apoplast comprising xylem, intercellular spaces and vascular interconnections (Hallmann 2001; Sattelmacher 2001; Bacon *et al.* 2002; Evert 2006; Compant *et al.* 2008, 2011). The intracellular bacterial association observed here is similar to the association documented with different insect species. Such associations have been symbiotic with the bacterium getting housing and nutrition from the host, and in turn the bacteria providing the host with essential vitamins, metabolites, toxin or other biochemicals that are either beneficial or essential to the host (McFall-Ngai 2002; Dale and Moran 2006; Wernegreen 2012).

The concept of intracellular colonization assumes importance in several respects. It is a priority task now to elucidate how the organisms gain access to the intracellular niche. Plasmodesmata are too small to allow the cell-to-cell movement of bacteria, unlike viruses (Epel 1994; Evert 2006). Endophytic bacteria are known to bear cellulolytic enzymes facilitating their selective recruitment by the host from environmental flora discriminating from potential plant pathogens (Rosenblueth and Martinez-Romero 2006; Compant *et al.* 2010). Once entry is gained, they could perpetuate further through the cell division cycle, particularly in a vegetatively propagated crop like banana. The intracellular colonization also has implications in functional plant biology. The organisms survive in the intracellular environment akin to cellular organelles living on the host resources. Furthermore, the microbial products are released into the cytoplasm where they can possibly influence gene expression and the functioning of the host. In the absence of recognizing the microbial association, microbe-associated activities would masquerade as normal plant activities and would not be attributed to the associated organisms. The debate on cytokinin synthesis by plants versus plant-associated bacteria (Holland 1997) is an example of this.

Endophytes are generally considered to be selected or recruited by the host plant from the rhizosphere (Hallmann *et al.* 1997; Rosenblueth and Martínez-Romero 2006; Compant *et al.* 2010). On the contrary, for clonally propagated plants, the established organisms become an integral component and there may be an endophytic continuum in tissue through generations (Thomas and Soly 2009; Compant *et al.* 2011; Thomas and Reddy 2013). Tissue culture, wherein the tissue could be kept away from possible contaminants, provides a good tool to study the perpetually shoot-associated organisms in organized tissue as well as *in vitro* derived unorganized tissue.

The primary focus in this study has been establishing whether the motile intracellular elements under simple high-magnification microscopy constituted bacterial cells. Aspects like how the organisms gained entry into the two intracellular niches, the diversity and the identity of the organisms, niche specificity, and above all, the functions of the organisms, need to be pursued to get a clear understanding of the role played by them in the biology of the host. Furthermore, the enormous number of organisms observed with bananas in whole plants and *in vitro* stocks suggests the possibility of interference from the associated organisms during molecular investigations. Molecular studies on plants generally rely on whole-tissue DNA without any consideration of endophytic microbial colonization. It is to be anticipated that DNA from such organisms could be purified with plant DNA preparations. It would, however, not be easy to eliminate all the organisms even with antibiotic treatments (Thomas 2011). The present observations thus open up new research opportunities and also possible applications by exploiting integrally plant-associated organisms in enhancing plant growth or as potential bio-control agents. These aspects would constitute future research topics.

## Conclusions

This study employing live cell imaging on fresh shoot tissue as well as cell cultures and protoplast systems with intact cell wall or cell membrane clearly demonstrates abundant intracellular bacterial inhabitation in two niches, namely in the cytoplasm and in the cell wall–plasma membrane peri-space. The observations, supported by direct bright-field microscopy and with the use of different bacterial stains under bright-field as well as fluorescence microscopy, prove that the motile intracellular particles generally observed in non-fixed fresh tissue also include bacteria besides the normal cell organelles. The organisms in the above two niches are designated as ‘Cytobacts’ and ‘Peribacts’ to enable their further detailed analysis and characterization. The observations with different genotypes and suckers obtained from geographically distant locations and the *in vitro* cultures initiated and maintained independently at different laboratories suggest the association is ubiquitous to banana genotypes. The integral and inseparable intracellular bacterial presence in substantial numbers in field plants and *in vitro* cultures has considerable implications in the biology of the host.

## Sources of Funding

This study was supported under the ICAR-AMAAS (Application of Microorganisms in Agriculture and Allied Sectors) project: ‘Basic and applied investigations on endophytic microorganisms in horticultural crops’. This publication bears IIHR contribution No. 117/2013.

## Contributions by the Authors

P.T. planned the experiments and executed the microscopy work. A.C.S. supported the sample preparation, *in vitro* culture initiation and maintenance, and sample preparations for microscopy. P.T. prepared the manuscript.

## Conflicts of Interest Statement

None declared.

## Supporting Information

The following Supporting Information is available in the online version of this article –

**Figure S1.** Confocal *z*-stacks of banana cv. Grand Naine embryogenic cell line after SYTO-9 staining showing abundant cytoplasmic and periplasmic bacteria in different cell planes over 30 µm depth at 1 µm intervals.

**Movie 01.** An *x*–*y*–*z*–*t* bright-field scan of fresh tissue section from leaf sheath of banana cv. Grand Naine showing actively motile micro-particles in the intracellular matrix (×100 objective).

**Movie 02.** Pure culture of endophytic *Enterobacter cloacae* under bright-field displaying active cell motility in a thin film of water (×100 objective).

**Movie 03.** An *x*–*y*–*z*–*t* bright-field scan of a fresh tissue section from leaf sheath of banana cv. Mysore under bright-field showing actively motile micro-particles in the intracellular matrix (×100 objective).

**Movie 04.** An *x*–*y*–*z*–*t* bright-field scan of fresh leaf-sheath sections from the shoots of cv. Grand Naine treated with TTC showing light tissue pigmentation along with pink-stained or unstained micro-particles in the intracellular matrix either as non-motile units or as actively mobile ones (×100 objective).

**Movie 05.** A view of the *x*–*y*–*t* bright-field scan of fresh tissue sections of banana cv. Grand Naine showing motile bacterial cells in the intracellular matrix after staining with dilute safranin (0.005 %) (×100 objective).

**Movie 06.** An *x*–*y*–*t* scan of callus cell from banana cv. Robusta displaying abundant organelles or inclusions distributed in the cell lumen and motile bacteria (×100 objective).

**Movie 07.** Isolated cell from embryonic cultures of banana cv. Grand Naine separated following enzymatic treatment confirming abundant cytoplasmic bacterial inhabitation (×100 objective).

**Movie 08.** Intravacuolar motile bacteria in enzyme-permeabilized cells stained with dilute safranin (×100 objective).

**Movie 09.** Confocal imaging on fresh ECS cells after SYTO-9 treatment showing the staining of periplasmic and cytoplasmic bacteria in different cell layers in the *x*–*y*–*z* scan (×63 objective). Video file generated from 30 s confocal scans with Image J showing short-span video.

**Movie 10.** Confocal imaging of fresh tissue sections after the enzymatic permeabilization treatment showing the staining of periplasmic and cytoplasmic bacteria with SYTO-9 in different cell layers in the *x*–*y*–*z* scan (×63 objective). Video file generated from 30 s confocal scans with Image J showing short-span video.

Additional Information
