# Supplementary material for: Live cell imaging reveals extensive intracellular cytoplasmic colonization of banana by normally non-cultivable endophytic bacteria
Source: AoB Plants. 2014 Jan 16;6:plu002. doi: 10.1093/aobpla/plu002 (PMC4038436; doi:10.1093/aobpla/plu002)
Supplement: Additional Information [file supp_6_plu002_index.html]

Live cell imaging reveals extensive intracellular cytoplasmic colonization of banana by normally non-cultivable endophytic bacteria — Live cell imaging reveals extensive intracellular cytoplasmic colonization of banana by normally non-cultivable endophytic bacteria — Additional Information 

# Live cell imaging reveals extensive intracellular cytoplasmic colonization of banana by normally non-cultivable endophytic bacteria

## Additional Information

Additional Information

**Files in this Data Supplement:**

- Additional Information - Doc file
- Additional Information Figure 1 - jpg file
- Additional Information Movie 1 - wmv file
- Additional Information Movie 2 - wmv file
- Additional Information Movie 3 - wmv file
- Additional Information Movie 4 - wmv file
- Additional Information Movie 5 - wmv file
- Additional Information Movie 6 - wmv file
- Additional Information Movie 7 - wmv file
- Additional Information Movie 8 - wmv file
- Additional Information Movie 9 - wmv file
- Additional Information Movie 10 - wmv file
