# Supplementary material for: Live cell imaging reveals extensive intracellular cytoplasmic colonization of banana by normally non-cultivable endophytic bacteria
Source: AoB Plants. 2014 Jan 16;6:plu002. doi: 10.1093/aobpla/plu002 (PMC4038436; doi:10.1093/aobpla/plu002)
Supplement: Additional Information [file supp_plu002_plu002supp.doc]

**SUPPORTING INFORMATION**

**Figure** **S1.** Confocal z-stacks of banana cv. Grand Naine embryogenic cell line after SYTO-9 staining showing abundant cytoplasmic and peri-space bacteria in different cell planes over 30 µm depth at 1 µm intervals.

**Movie Files**

**File 01.** Movie 01. An *x-y-z-t* bright-field-scan of fresh tissue section from leaf sheath of banana cv. Grand Naine showing actively motile micro-particles in the intra-cellular matrix (100x objective).

**File 02.** Movie 02. Pure culture of endophytic *Enterobacter cloacae* under bright-field displaying active cell motility in a thin film of water (100x objective).

**File 03.** Movie 03. An *x-y-z-t* bright-field-scan of fresh tissue section from leaf sheath of banana cv. Mysore under bright field showing actively motile micro-particles in the intra-cellular matrix (100x objective).

**File 04.** Movie 04. An *x-y-z-t* bright-field scan of fresh leaf-sheath sections from the shoots of cv. Grand Naine treated with triphenyl tetrazolium chloride showing light tissue pigmentation along with pink-stained or un-stained micro-particles in the intracellular matrix either as non-motile units or as actively mobile ones (100x objective).

**File 05.** Movie 05. A view of *x-y-t* bright-field scan of fresh tissue-sections of banana cv. Grand Naine showing motile bacterial cells in the intracellular matrix after staining with dilute safranin (0.005%) (100x objective).

**File 06.** Movie 06. An *x-y-t* scan of callus cell from banana cv. Robusta displaying abundant organelles or inclusions distributed in the cell lumen and motile bacteria (100x objective).

**File 07.** Movie 07. Isolated cell from embryonic cultures of banana cv. Grand Naine separated following enzymatic treatment confirming abundant cytoplasmic bacterial inhabitation (100x objective).

**File 08.** Movie 08. Intra-vacuolar motile bacteria in enzyme permeabilized cells stained with dilute safranin (100x objective).

**File 09.** Movie 09. Confocal imaging on fresh ECS cells after SYTO-9 treatment showing the staining of periplasmic and cytoplasmic bacteria in different cell layers in *x-y-z* scan (63x objective). Video file generated from 30 s confocal scans with Image J showing short-span video.

**File 10.** Movie 10. Confocal imaging of fresh tissue sections after the enzymatic permeabilization treatment showing the staining of periplasmic and cytoplasmic bacteria with SYTO-9 in different cell layers in *x-y-z* scan (63x objective). Video file generated from 30 s confocal scans with Image J showing short-span video.
