# Supplementary figures and images for: Live cell imaging reveals extensive intracellular cytoplasmic colonization of banana by normally non-cultivable endophytic bacteria
Source: AoB Plants. 2014 Jan 16;6:plu002. doi: 10.1093/aobpla/plu002 (PMC4038436; doi:10.1093/aobpla/plu002)

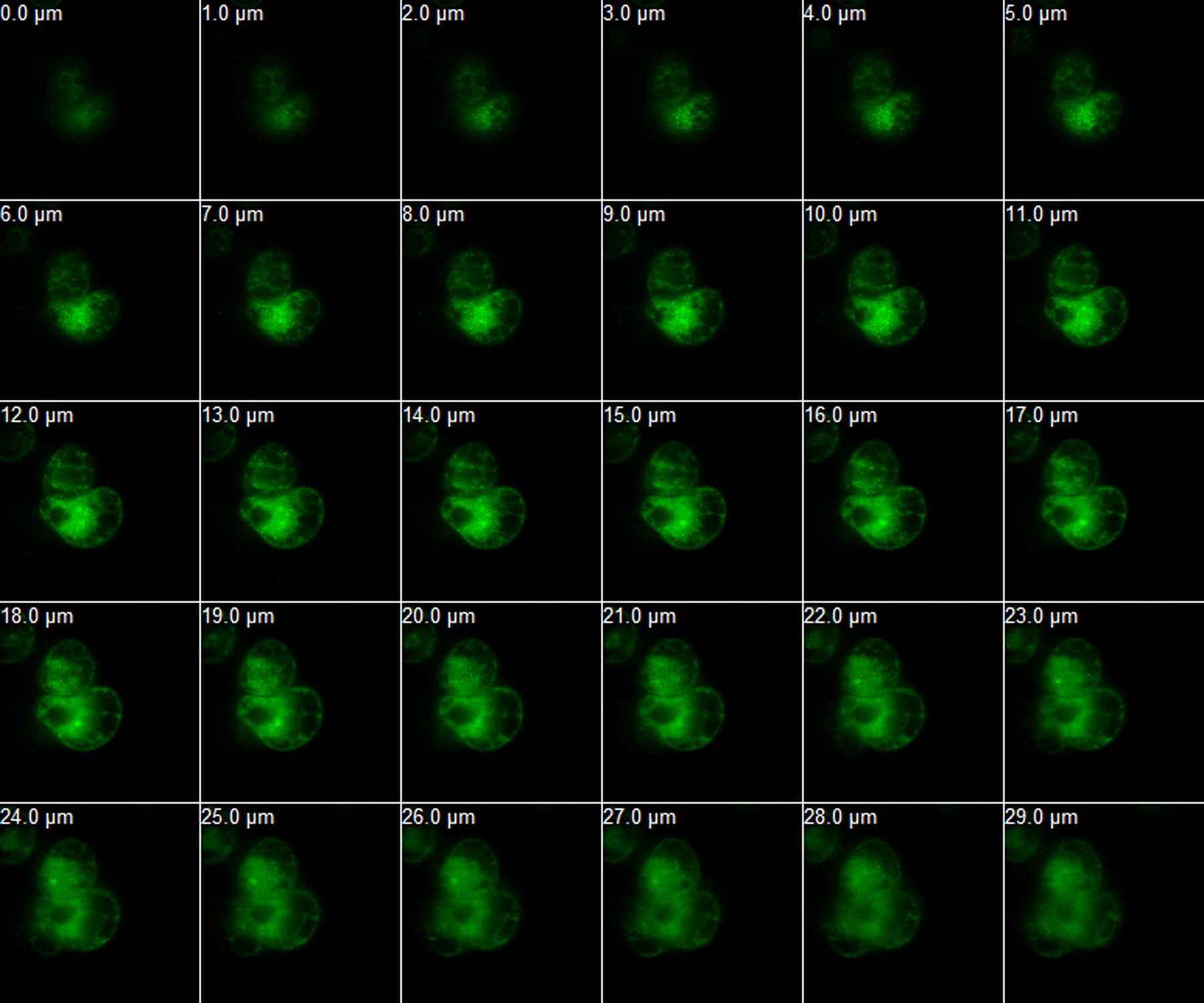

Supplement: Additional Information [file supp_plu002_plu002supp_figure1.jpg]
